# Exploring Microphase Separation in Semi-Fluorinated Diblock Copolymers: A Combined Experimental and Modeling Investigation

**DOI:** 10.1021/acspolymersau.5c00109

**Published:** 2025-10-10

**Authors:** Mona Semsarilar, Martin J. Greenall, Alex H. Balzer, Amit Kumar Sarkar, Chaimaa Gomri, Belkacem Tarek Benkhaled, Anke-Lisa Höhme, Martin Held, Volker Abetz, Helena J. Hutchins-Crawford, Georgia L. Maitland, Anisha Patel, Thomas H. Epps, Paul D. Topham, Matthew J. Derry

**Affiliations:** † Institut Européen des Membranes (IEM), CNRS, ENSCM, 119128 Univ Montpellier, Montpellier 34090, France; ‡ School of Engineering and Physical Sciences, University of Lincoln, Brayford Pool, Lincoln LN6 7TS, U.K.; § Center for Plastics Innovation (CPI), 5972 University of Delaware, Newark, Delaware 19716, United States; ∥ Department of Chemical and Biomolecular Engineering, University of Delaware, Newark, Delaware 19716, United States; ⊥ Department of Chemical Engineering and Biotechnologies, College of Engineering and Physical Sciences, 1722 Aston University, Birmingham B4 7ET, U.K.; # 28338 Helmholtz-Zentrum Hereon, Institute of Membrane Research, Max-Planck-Straße 1, Geesthacht 21502, Germany; ∇ Institute of Physical Chemistry, University of Hamburg, Grindelallee 117, Hamburg 20146, Germany; ○ Aston Institute for Membrane Excellence, Aston University, Birmingham B4 7ET, U.K.; ◆ Center for Research in Soft matter and Polymers (CRiSP), University of Delaware, Newark, Delaware 19716, United States

**Keywords:** block copolymers, self-assembly, strong segregation theory, X-ray scattering, X-ray reflectometry

## Abstract

We report the combined experimental and theoretical study of the bulk self-assembly behavior of polystyrene-*block*-poly­(2,3,4,5,6-pentafluorostyrene) diblock copolymers. These block copolymers were designed to create highly antagonistic blocks (with a high Flory–Huggins interaction parameter, χ) with minimum disruption to the molecular construct (i.e., only replacing five hydrogen atoms with five fluorine atoms). A large library of diblock copolymers (41 samples) was synthesized by reversible addition–fragmentation chain transfer (RAFT) polymerization to map out a major portion of the phase space. All block copolymers exhibited narrow molecular weight distributions with dispersity (*D*) values between 1.07 and 1.32, and subsequent thermal annealing revealed phase separation into well-defined nanoscale morphologies depending on their molecular composition, as determined from small-angle X-ray scattering and transmission electron microscopy analyses, with an experimental phase diagram being constructed. The χ value at 25 °C for this block copolymer was estimated to be 0.2 using strong segregation theory, based on trends in phase-separated domain spacing and interfacial width. When applying theoretical approaches, the majority of the domain spacing data trends were captured by a coil–coil diblock copolymer model; however, a better fit to the data for samples with shorter fluorinated blocks was obtained with a rod–coil model, indicating that the chains in these fluorinated blocks likely have a higher inherent stiffness and were thus rod-like. This observation demonstrates that, due to the very high value of χ, a transition from coil–coil to rod–coil behavior can be obtained purely by reducing the length of the stiffer of the two blocks and without varying temperature or the chemical composition of the polymers. This work showcases the presence of strong microphase separation within AB diblock copolymers despite the relatively similar chemical composition of the constituent “A” and “B” units, with a clear transition from rod–coil to coil–coil segregation behavior.

## Graphical abstract

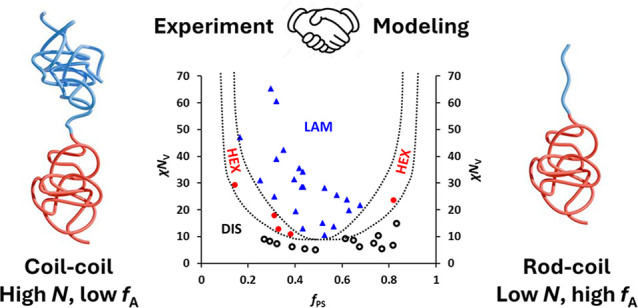

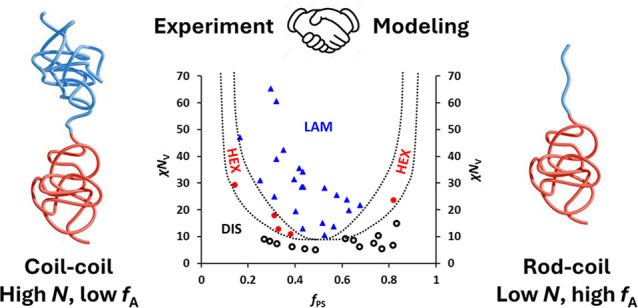

## Introduction

Advances in “living” polymerization techniques such as anionic polymerization and reversible-deactivation radical polymerization (RDRP)^1^ methods, including reversible addition–fragmentation chain transfer (RAFT) polymerization,^2^ atom transfer radical polymerization (ATRP),^3,4^ and nitroxide-mediated polymerization (NMP),^5^ have enabled the facile synthesis of polymers with well-defined molecular architectures. Among the most studied of these architectures is block copolymers,^6^ which are macromolecules containing two chemically distinct segments, or “blocks”, that are covalently bonded, such as in an AB diblock copolymer. Due to the inherent incompatibility between the “A” and “B” segments, many AB diblock copolymers undergo nanoscale phase separation in the bulk and can adopt various nanostructures, e.g., spherical, gyroid, hexagonally packed cylindrical, or lamellar morphologies.^7^ Such self-assembly in block copolymers is governed by parameters such as the volume fraction of the “A” block (*f*_A_), total number of monomer segments in the block copolymer (*N*), and Flory–Huggins interaction parameter (χ).^7,8^ Variables *f*_A_ and *N* are controlled synthetically, whereas χ is a measure of the thermodynamic incompatibility in the system (i.e., between the “A” and “B” repeating units). AB diblock copolymers can undergo spontaneous self-assembly when the product χ*N* is ≥10.495.^9^

Theoretical phase diagrams can be generated using techniques such as self-consistent field theory (SCFT) to predict the nanomorphology adopted by a given block copolymer,^7,10,11^ and experimental polymer scientists often strive to construct phase diagrams to map the self-assembly behavior of block copolymers and target desired morphologies.^12–16^ Such understanding of the spontaneous molecular organization into well-defined nanostructures lends these materials to various applications in soft electronics, data storage, energy storage, separation science, etc.^7^ Key to enabling the construction of experimental phase diagrams is knowledge of χ for a given AB diblock copolymer. χ values can be estimated both experimentally (e.g., using small-angle X-ray scattering (SAXS) or rheological studies) and computationally (e.g., SCFT or strong segregation theory (SST)), or a combination of the two. SAXS and X-ray reflectivity (XRR) measurements are effective and nondestructive techniques that allow assessment of the degree of mixing by evaluating the interfacial width in block copolymer systems. Larger χ values indicate a stronger propensity for phase separation, meaning that self-assembly can be achieved with low molar mass block copolymers, i.e., those with lower *N* values, which paves the way for the generation of morphologies with extremely small domain sizes. Consequently, there is a collective quest for so-called high χ-low *N* block copolymers, which have particular promise in nanolithography applications^17,18^ and membrane technologies,^19^ among other areas. However, many commonly studied AB diblock copolymers do not have intrinsically high χ values; for example, poly­(methyl methacrylate)-*b*-polystyrene has a χ value of 0.043 at 22 °C,^20^ in which case comparatively high *N* values are required to induce strong segregation and thus larger domain sizes are formed. Consequently, researchers have employed various methods to induce increased incompatibility between the A and B blocks, including using monomers with electron-dense atoms.

The incorporation of repeat units with highly contrasting chemical functionality within an AB diblock copolymer is one approach to induce high degrees of incompatibility, which often facilitates the generation of long-range ordering with fewer defects and sharper interfaces, enabling the use of these materials in practical thin-film applications that require precisely controlled domains and channels such as in nanolithography.^17,18^ Enhanced phase separation can be achieved by subtly modifying the chemical structure of a repeating unit. For example, block copolymers of polystyrene and poly­(2-vinylpyridine) or poly­(4-vinylpyridine) show relatively distinct levels of thermodynamic incompatibility despite the minimal structural difference between these residues.^21–24^ Introducing fluorine atoms to monomer repeat units via postpolymerization functionalization is also an effective way of generating block copolymers with high χ values, as has been demonstrated by Hillmyer, Lodge and coworkers^25–30^ and recently by Patel et al.^31^ and Yang et al.^32^ For example, fluorination of butadiene units in polystyrene-*block*-poly­(1,2-butadiene) has been shown to induce microphase separation even when the precursor AB diblock copolymers are disordered.^30^ Other strategies to generate high χ values in semifluorinated block copolymers have involved the use of partially fluorinated (meth)­acrylate-based or styrene-based^33^ polymer blocks in combination with polystyrene.^33–36^ Microphase separation in such high-χ polymers can be modeled effectively by SST,^37,38^ which allows the value of the χ parameter to be determined by comparing experimental results for the dependence of the microphase domain spacing and interfacial width on the degree of polymerization with the corresponding theoretical predictions. The interfacial width is a measure of how sharp or diffuse the interface is between constituent microdomains.^39–41^ It is intrinsically related to the value of χ and the degree of segregation. In the strong segregation limit (SSL, wherein χ*N* ≫ 10.495), the interfacial width is reduced because the high repulsion between different blocks minimizes their intermixing at the interface, resulting in sharp, well-defined boundaries.^42^

Herein, we report a series of polystyrene-*b*-poly­(2,3,4,5,6-pentafluorostyrene) [PS-*b*-PPFS] AB diblock copolymers synthesized via RAFT polymerization. These semifluorinated block copolymers serve as a model system in which the only difference between the constituent monomer repeating units is the substitution of five hydrogen atoms with five fluorine atoms. This relatively subtle difference in chemical structure between the A and B blocks drives strong phase segregation in the system, as evidenced by the formation of well-ordered domains observed through SAXS experiments. SAXS and XRR enabled estimation of the χ value for this block copolymer and the domain spacing trends adopted by this model system, and SST supports our experimental observations. Moreover, this study demonstrated that simply substituting only five atoms in one block facilitated the generation of highly segregated diblock copolymers with an estimated χ value of 0.2, controlled morphologies, and small domain sizes. SST also allowed investigation of whether PS-*b*-PPFS behave as coil–coil and rod–coil chains. Although the term “rod–coil” does not align with the labeling of the diblock copolymers as PS-*b*-PPFS, because it is the PPFS block that is treated as a rod, we use this terminology instead of “coil–rod” due to its wider adoption in the literature.^43–48^ The high value of χ means that self-assembly can still occur when one of the two blocks is too short to be modeled as a coil, making the molecules behave as rod–coil polymers.^13^ Despite the large entropic penalty associated with such a short “rod” self-assembling into its own domain, the counter-acting enthalpic driving force of demixing (due to the high χ value) overcomes this, leading to phase separation. The large range of phase space covered in this study reveals that a transition from coil–coil to rod–coil behavior can be controlled simply by tuning the degree of polymerization of the stiffer PPFS block rather than, as in previous work, varying temperature,^43,49,50^ chemical composition of the rod block,^51^ or solvent conditions.^44^ Applying both coil–coil and rod–coil^45^ versions of the theory enables elucidation of the coil–coil to rod–coil transition in this PS-*b*-PPFS system.

## Materials and Methods

### Materials

2,3,4,5,6-Pentafluorostyrene (PFS) was purchased from Apollo Scientific (UK). Styrene, 2,2′azobis­(isobutyronitrile) (AIBN), 2-(dodecylthiocarbonothioylthio)-2-methylpropionic acid (DDMAT), toluene, methanol, and deuterated solvents for ^1^H nuclear magnetic resonance (NMR) analysis were purchased from Sigma-Aldrich (France). ACS Optima grade tetrahydrofuran (THF) used in the preparation of thin films for XRR was purchased from Fisher Scientific (USA). All materials were used as received.

### Synthesis of PS Macromolecular Chain Transfer Agent (Macro-CTA) via RAFT Solution Polymerization

A typical protocol for the synthesis of a PS macro-CTA was as follows. DDMAT RAFT agent (0.875 g, 2.40 mmol), AIBN initiator (0.079 g, 0.50 mmol, CTA/AIBN molar ratio = 5.0), styrene monomer (10.0 g, 0.096 mol; target degree of polymerization, DP = 40) and toluene (10.0 g) were weighed into a 25 mL round-bottom flask. The reaction flask was placed in an ice bath, and the solution was purged with nitrogen for 15 min. The sealed flask was immersed into an oil bath set at 70 °C for 8 h (final styrene conversion = 50%, as judged by ^1^H NMR spectroscopy when comparing the peaks for vinyl protons from unreacted monomer with aromatic protons from PS, see Figure S1a), and the polymerization was subsequently quenched by immersion in liquid nitrogen. THF (15 mL) was added to the reaction solution, followed by precipitation into a 10-fold excess of cold methanol (250 mL). The precipitated PS macro-CTA was redissolved in THF, and the precipitation was twice repeated. The separated polymer was then dried under vacuum for 17 h. For this representative PS macro-CTA, ^1^H NMR analysis of the purified PS macro-CTA indicated a mean degree of polymerization of 21 by comparing the peaks for aliphatic protons from the chain-end with aromatic protons from PS (see Figure S1b), and gel permeation chromatography (GPC) analysis (Figure S2) indicated a number-average molar mass (*M*_n_) of 2,700 g mol^–1^ and a molar mass dispersity (*D*) of 1.07. Refer to Table S1 for full molecular characterization of all PS macro-CTAs synthesized.

### Synthesis of PS-*b*-PPFS Diblock Copolymer via RAFT Solution Polymerization

A typical protocol for the synthesis of PS_21_-*b*-PPFS_27_ diblock copolymer was as follows. PS_21_ macro-CTA (0.23 g, 0.11 mmol), AIBN (5.44 mg, 0.03 mmol, macro-CTA/AIBN molar ratio = 3.6), PFS monomer (0.60 g, 3.10 mmol; target DP = 28) and toluene (0.60 g) were weighed into a 5 mL round-bottomed flask. The sealed flask was then placed in an ice bath and degassed for 5 min prior to immersion in an oil bath set at 70 °C for 24 h. The conversion of the PFS monomer was 97% as judged by ^19^F NMR spectroscopy when comparing the peaks from unreacted monomer to those from PPFS (see Figure S3). The reaction was then quenched removing from the oil bath to cool and exposing to air. The resulting polymer was purified by twice precipitating into cold methanol. The obtained yellow powder was then dried under vacuum for 17 h and further analyzed by ^1^H NMR spectroscopy (see Figure S4). Refer to Table S1 for full molecular characterization of all PS-*b*-PPFS polymers synthesized.

### NMR Spectroscopy

NMR spectra were recorded on a Bruker AV III HD Spectrometer (400 MHz for ^1^H and 376 MHz for ^19^F). The samples were dissolved in CDCl_3_ or CD_2_Cl_2_ before analysis. The experimental conditions for recording ^1^H and ^19^F NMR spectra were as follows: flip angle, 90° (or 30°); acquisition time, 4.5 s (or 2 s); pulse delay, 2 s; number of scans, 32 (or 64); and pulse widths of 12.5 and 11.4 μs for ^1^H and ^19^F NMR spectroscopy, respectively.

### Gel Permeation Chromatography (GPC)

Polymer molar mass distributions were analyzed using a Viscotek TDA 305 instrument fitted with 2 PolarGel M 300 × 7.5 columns thermostated at 35 °C and a refractive index detector. The mobile phase was THF containing 0.3% w/w toluene at a flow rate of 1 mL min^–1^. The calibration was performed using near-monodisperse PS standards with *M*_p_ ranging from 400 to 12,000 g mol^–1^ (Malvern).

### Thermogravimetric Analysis (TGA)

TGA was used to assess the thermal degradation temperature of PS-*b*-PPFS by monitoring the relative change in mass as a function of increasing temperature. TGA was performed using a PerkinElmer Pyris 1 thermogravimetric analyzer under nitrogen atmosphere (flow rate 20 mL min^–1^). Samples were heated from 25 to 600 °C at a rate of 10 °C min^–1^.

### Small-Angle X-ray Scattering (SAXS)

SAXS patterns were recorded at Diamond Light Source using a Xeuss 3.0 SAXS instrument (Xenocs, France) equipped with a liquid gallium and indium alloy MetalJet X-ray source (Excillum, Sweden, λ = 0.134 nm) operating at 70 kV, two sets of motorized scatterless slits for beam collimation and an Eiger2 R 1 M pixel detector (sample-to-detector distance = 1.00 m). SAXS patterns were recorded from *q* = 0.07 nm^–1^ to *q* = 3.0 nm^–1^, for which *q* = (4π sin θ)/λ is the length of the scattering vector and θ is one-half of the scattering angle. Glass capillaries of 1.5 mm diameter were used as sample holders and patterns were recorded and averaged over four 5 min periods. Data were reduced (normalized, integrated and averaged) using standard routines available at the beamline.^52,53^

### Transmission Electron Microscopy (TEM)

Bulk polymer films were prepared by casting a 60 g L^–1^ polymer solution in chloroform into a polytetrafluoroethylene (PTFE) vial and letting it slowly dry in a chloroform-saturated atmosphere at 23 °C for 21 days. The dried polymer was annealed in the PTFE vial for 24 h at 100 °C and subsequently for 2 h at 130 °C under vacuum, i.e., above the glass transition temperature (*T*_g_) of PS. The brittle films were removed from the vial by casting an embedding epoxy (Epo-Tek 301) on top, followed by embedding once more to stabilize the brittle polymer during subsequent trimming and microtomy. After slicing the stack with a diamond wire saw, polymer thin films were sectioned via ultramicromy with a Leica Ultracut at 20 °C. Due to the sectioned film’s brittle nature, the nominal thickness of 50 nm varied within the thin section. TEM images were recorded in bright field mode using a Tecnai G2 F20 (FEI Thermo Fisher Scientific) microscope operating at an accelerating voltage of 120 kV, equipped with a Gatan OneView Camera for high-resolution imaging.

### X-ray Reflectometry (XRR)

Thin films were cast onto UV-ozone cleaned silicon substrates via flow coating^54^ from 5% w/w solutions of polymer in THF. Films of uniform thickness were produced in constant velocity mode. Film thicknesses were measured using a reflectance spectrometer (Filmetrics F20–UV). The films were dried under dynamic vacuum at 22 °C for 18 h and subsequently annealed under static vacuum at 150 °C for 1 h. XRR was performed on an Ultima IV unit (Rigaku) at 22 °C with a thin, parallel beam of Cu Kα radiation, λ = 0.154 nm, incident on the quenched samples. The beam was sized to capture the critical edge of the samples for best results and fit accuracy. XRR profiles were collected by scanning a small incident angle (θ) of X-rays from the source and a detection angle (2θ) of reflected X-rays (0° < 2θ < 3°). Optimization and refinement to achieve final densities, film thickness, and roughness parameters then were performed using GlobalFit software. Surface roughness (*R*_*s*_) values were calculated using *R*_*s*_ = (2π)^1/2^δ ≈ 2.5δ, wherein δ is the average layer roughness from the model fits.

### Strong Segregation Theory (SST)

SST^37^ is a mean-field model that predicts the domain spacing (or spatial period) for simple ordered phases in melts of coil–coil block copolymers as a function of χ and the degree of polymerization *N*_V_, which is measured here in units of the segment volume of PS, 1/ρ_0PS_. SST assumes that the interfacial width over which the polymer composition transitions from one block type to another is small compared to the domain spacing, which occurs when the product χ*N*_V_ is sufficiently high. An extended version of SST^38,42^ also predicts the interfacial width, *t*_i_, and is more accurate when the interface is relatively broad (ideally, *t*_i_ should be much greater than the statistical segment length divided by $\sqrt{6}$ , although it should remain small compared to the domain spacing.^37,38^ The additional restriction used when predicting the interfacial width means that the SST predictions for *t*_i_ are likely to have a smaller region of validity than those for the domain spacing. The ratio of *t*_i_ to the domain spacing *d*, in the lamellar phase is given in this extended SST by^38,42^

$${\left(\frac{t_{i}}{d}\right)}_{\mathrm{SST}}={\left(\frac{\pi }{2\sqrt{6}\chi N_{V}}\right)}^{2/3}\left[1+\frac{4}{\pi }{\left(\frac{3}{\pi^{2}\chi N_{V}}\right)}^{1/3}\right]$$ 1

For calculations using SAXS and XRR data, χ is denoted as χ_eff_ (effective χ) to reflect the experimental data that may capture real-system effects such as thermal fluctuations and morphological defects. When fitting the domain spacing data, we modify the original SST model of domain spacings to allow for a difference in statistical segment lengths between the two blocks by using an asymmetry parameter^55,56^

2 $$\epsilon =\frac{a_{\mathrm{PS}}^{2}{\rho_{0}}_{\mathrm{PS}}}{a_{\mathrm{PPFS}}^{2}{\rho_{0}}_{\mathrm{PPFS}}}$$

wherein *a*_PS_ and *a*_PPFS_ are the statistical segment lengths of the two species, and ρ_0PS_ and ρ_0PPFS_ are the respective segment densities. The formula for *d* of lamellae then becomes

3 $$d=2{\left(\frac{4\gamma a_{\mathrm{PS}}^{2}}{\pi^{2}{\rho_{0}}_{\mathrm{PS}}}\right)}^{1/3}\frac{1}{{\left(\epsilon f_{\mathrm{PPFS}}+1-f_{\mathrm{PPFS}}\right)}^{1/3}}N_{V}^{2/3}$$

wherein γ is the surface tension (which can be related to χ), and *f*_PPFS_ is the volume fraction of PPFS. If ϵ = 1, such that there is no conformational asymmetry, the original SST is recovered. If ϵ → 0 (which means that the statistical segment length of PPFS is sufficiently long for this block to be treated as a rod), a formula for the domain spacing of rod–coil block copolymers in the lamellar phase developed by Müller and Schick is found:^45^

4 $$d=2{\left(\frac{4\gamma a_{\mathrm{PS}}^{2}}{\pi^{2}{\rho_{0}}_{\mathrm{PS}}}\right)}^{1/3}\frac{1}{{\left(1-f_{\mathrm{PPFS}}\right)}^{1/3}}N_{V}^{2/3}$$

## Results and Discussion

### Block Copolymer Synthesis and Characterization

PS-*b*-PPFS block copolymers were synthesized via two RAFT solution polymerization steps, each conducted in toluene (see Scheme 1).

**Scheme 1:**
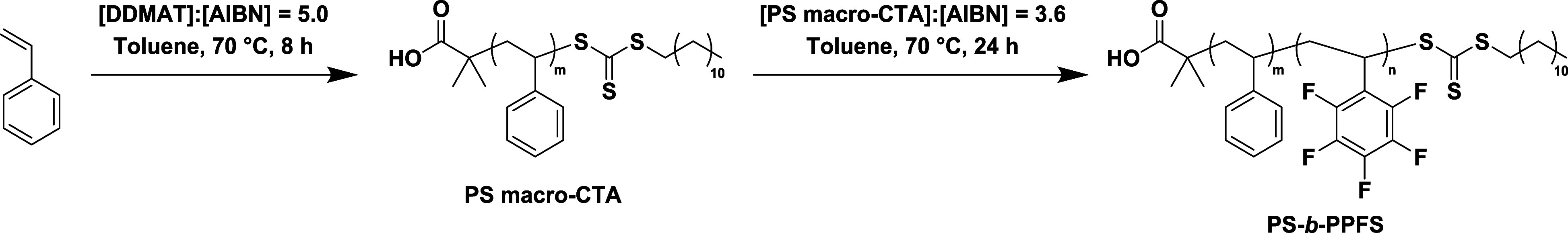
Synthesis of PS Macro-CTA via RAFT Solution Polymerization in Toluene at 70 °C, Followed by Chain Extension via RAFT Solution Polymerization of 2,3,4,5,6-Pentafluorostyrene (PFS) in Toluene at 70 °C to Yield PS-*b*-PPFS Diblock Copolymers

First, a series of PS macro-CTAs was synthesized using DDMAT as the RAFT agent, with target DPs ranging from 20 to 190 (see Table S1). ^1^H NMR spectroscopy analyses (see Figure S1a) confirmed that intermediate styrene monomer conversions between 50% and 73% were achieved after 8 h for these syntheses, which is important to ensure high chain-end fidelity and maximize efficiency in subsequent block copolymer syntheses.^57^ Unreacted monomer was removed via precipitation into cold methanol, and purified homopolymers were characterized using ^1^H NMR spectroscopy (see Figure S1b), which confirmed the synthesis of PS macro-CTAs (and successful removal of styrene monomer) with DP values ranging from 12 to 97. Additionally, good control over the RAFT solution polymerization during the preparation of these macro-CTAs was confirmed using GPC (see Figure S2), with *Đ* values ≤1.16 in all cases. Subsequently, each PS macro-CTA was used to prepare PS-*b*-PPFS diblock copolymers via additional RAFT solution polymerizations in toluene with target PPFS DPs ranging from 5 to 300 (see Table S1). ^1^H and ^19^F NMR spectroscopy analyses (see Figure S3) indicated that high PFS monomer conversions were achieved within 24 h in most cases; however, intermediate conversions were obtained in some block copolymer synthesis steps (e.g., 44% when targeting PS_97_-*b*-PPFS_300_). In the context of this study, incomplete monomer conversion during block copolymer synthesis is not problematic because unreacted monomer was subsequently removed via precipitation into cold methanol to isolate purified PS-*b*-PPFS. Most importantly, the actual PPFS DP obtained was determined using ^1^H NMR spectra obtained for the purified PS-*b*-PPFS block copolymers (see Figure S4). GPC analysis (see Figure S5) confirmed the successful synthesis of well-defined PS-*b*-PPFS block copolymers, with all *Đ* values being ≤1.32. Summaries of all molecular characterization of polymers synthesized in this work can be found in Table S1.

### Block Copolymer Microphase Separation

The PS-*b*-PPFS block copolymers synthesized and purified as described above were then assessed for their bulk self-assembly behavior. The densities of PS and PPFS homopolymers were taken to be 1.05 g cm^–3^ and 1.55 g cm^–3^, respectively, as previously reported.^58,59^ For comparison with the literature and theoretical modeling of microphase separation, the total degree of polymerization for the block copolymers was normalized to the reference molecular volume of one styrene monomer residue, calculated from the density of PS to be 165 Å^3^. Taking the above density values and the experimentally determined DP values for each block, the volume fraction of each block (*f*_PS_ and *f*_PPFS_) and volume-normalized total degree of polymerization (*N*_V_) were calculated for each PS-*b*-PPFS block copolymer (see Table S1). Thermal annealing was conducted by heating at 150 °C for 1 h before cooling slowly to 20 °C. A maximum temperature of 150 °C was chosen for annealing because this temperature is above the *T*_g_ of both PS and PPFS polymer blocks and thus enables significant chain mobility, but importantly, significantly below the degradation temperature of the block copolymers as judged by thermogravimetric analysis (see Figure S6). Following this annealing step, the morphology of each PS-*b*-PPFS copolymer was determined using a laboratory SAXS instrument at Diamond Light Source, UK (see Figure 1). For many samples, microphase separation into well-ordered domains was evident by the presence of multiple isotropic rings in the 2D SAXS patterns (see Figure S7), which corresponded to sharp peaks in the 1D SAXS patterns (see Figure 1). In some cases, up to 7 or 8 peaks were identifiable for a single sample (e.g., PS-*b*-PPFS with *N*_V_ = 327, *f*_PS_ = 0.30 and *N*_v_ = 303, *f*_PS_ = 0.32; see in Figure 1), which is strongly indicative of the high degree of order in these block copolymers given that these data were acquired using a laboratory-based instrument, also aided by the high electron density contrast between the fluorinated and nonfluorinated domains. For samples exhibiting multiple peaks, morphology identification was conducted by comparing the position of peaks relative to the primary peak (q*), which is the peak at the lowest *q* value for each sample. Morphologies present in these PS-*b*-PPFS block copolymers were lamellar (LAM, with a *q*/q* peak sequence of 1, 2, 3, 4, .), hexagonally packed cylinders (HEX, with a *q*/q* peak sequence of 1, $\sqrt{3}$ , $\sqrt{4}$ , $\sqrt{7}$ , .), and disordered (DIS, with only one broad q* peak).^60^

**Figure 1:**
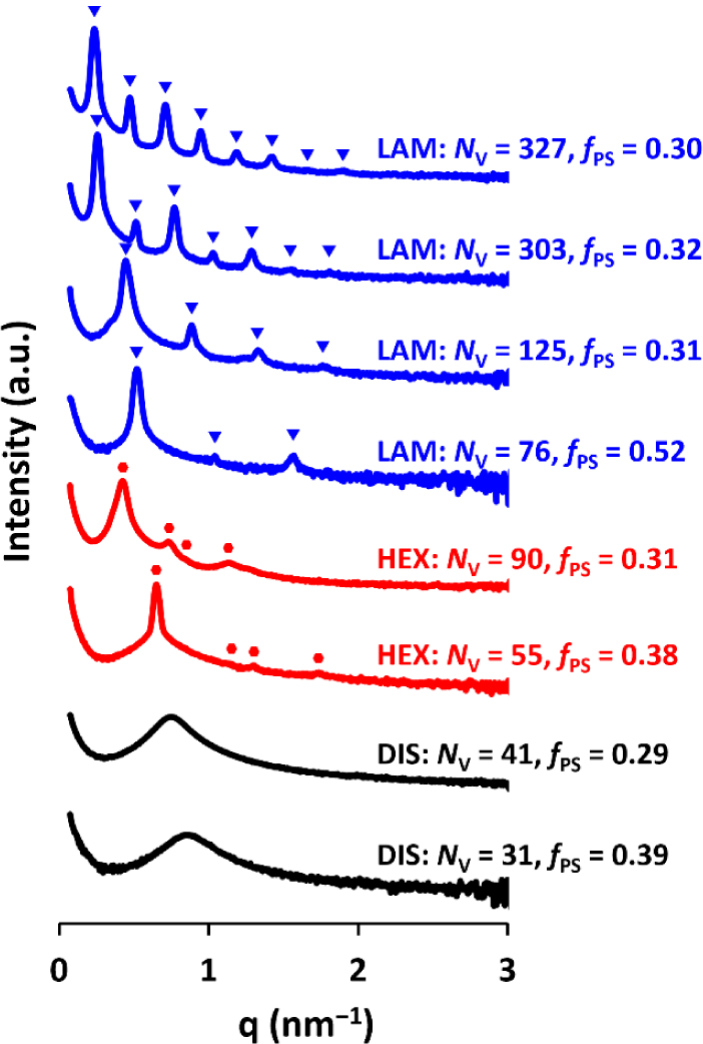
1D SAXS patterns recorded for selected PS-*b*-PPFS block copolymers with varying *N*_V_ and *f*_PS_. Blue triangles highlight peak positions for LAM and red circles for HEX.

Following SAXS analysis, PS-*b*-PPFS samples were prepared for imaging via TEM. PS-*b*-PPFS solutions were cast onto PTFE substrates and allowed to dry slowly at 23 °C over 21 days before being embedded into epoxy resin, sliced, and sectioned via ultramicrotomy to facilitate TEM imaging. Importantly, the TEM images obtained (Figure 2) support morphology assignments made using SAXS data. Because SAXS analysis is considered more statistically robust due to the facts that a much larger portion of the specimen is sampled and the data are not influenced by surface defects, SAXS was used as the primary technique to assign PS-*b*-PPFS morphologies.

**Figure 2:**
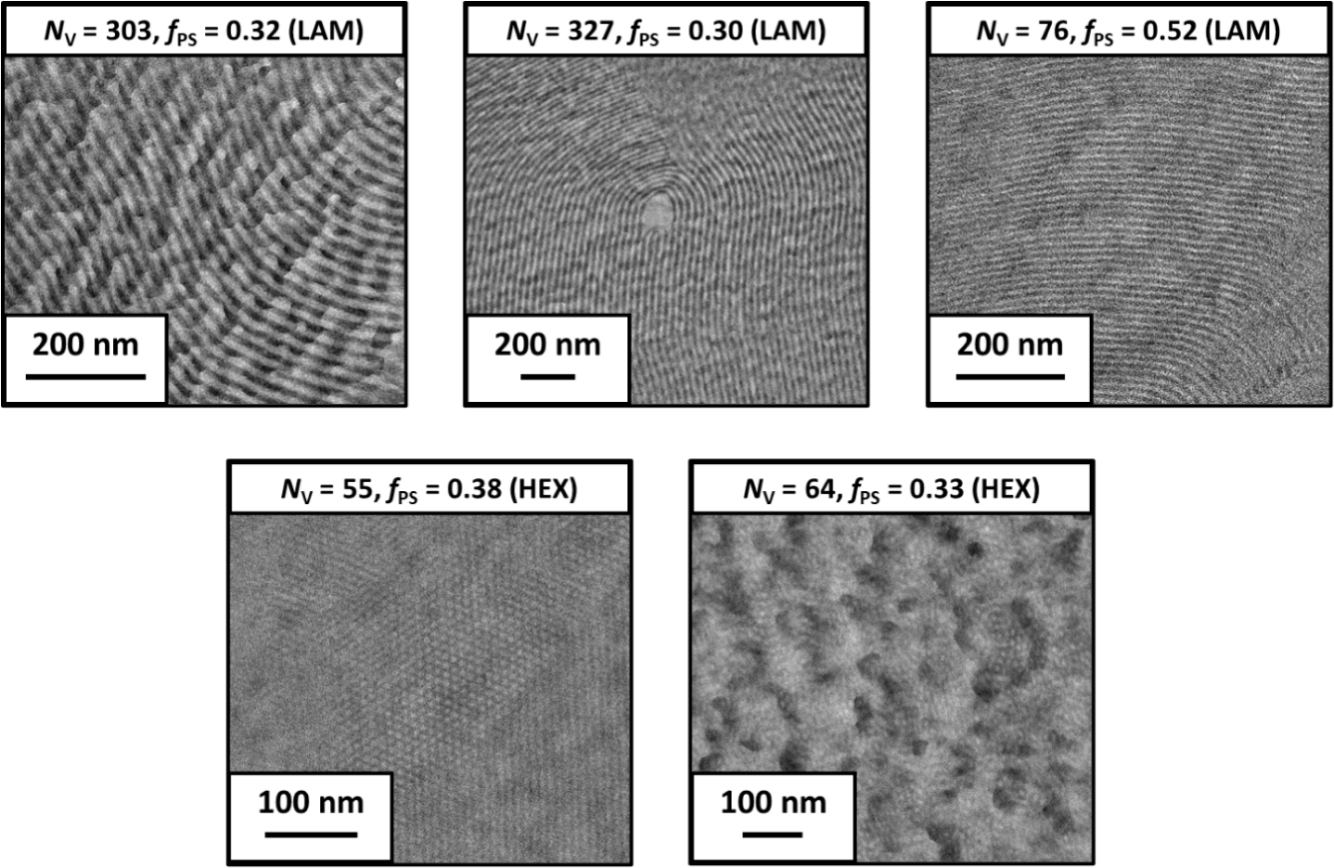
TEM images obtained for selected PS-*b*-PPFS block copolymers with varying *N*_V_ and *f*_PS_.

### Estimation of χ Using SAXS and XRR

Lamellar morphologies exhibited in copolymers with high *N*_V_ values permitted the measurement and calculation of interfacial width (along with the domain spacing) via SAXS and XRR (see Figure 3a). The block copolymers displayed an expected increase in *d* with increasing *N*_V_. Interfacial widths determined by SAXS were calculated using the method previously described by Register et al.,^42^ and χ_eff_ values were determined using eq 1 This relationship assumed that the SST predictions for *t*_i_ and *d* held true (see Supporting Information for detailed discussion). Over the range of *N*_V_ tested (53–327), χ_eff_ had an average of 0.16 ± 0.05. There was a slight decrease in χ_eff_ (Figure 3b) as *N*_V_ increased, which was more apparent at low *N*_V_. χ_eff_ ideally should only be a relation between the two monomer units and independent of *N*_V_; however, the monomers did not change in the block copolymers studied, and the change in χ_eff_ was likely due to the small *N*_V_ values, with comparatively small *t*_i_ values of these shorter polymers. More specifically, if the statistical segment length of polystyrene is 0.67 nm,^61^ the requirement for the predictions of SST for the interfacial width to be valid^37^ becomes $t_{i}\gg 0.67/\sqrt{6}=0.27\mathrm{nm}$ , which is not satisfied by our smallest interfacial widths, which are as low as 0.8 ± 0.1 nm. The calculation of interfacial width also uses the domain spacing result, which is not valid at small *N*_V_. Additionally, it is likely that there are deviations from a coil–coil to rod–coil chain morphology, further discussed in the next section. Values for *d* and *t*_i_ also were measured for four samples using XRR (Figure 3a). GlobalFit software was used to model the multilayered lamellar structures present on the silicon substrates. Each individual layer thickness was averaged and the sum of the average PS and PPFS layers used to determine *d*. There was good agreement between the *d* and *t*_i_ values determined by SAXS and XRR at high *N*_v_ (see Table S2).

**Figure 3:**
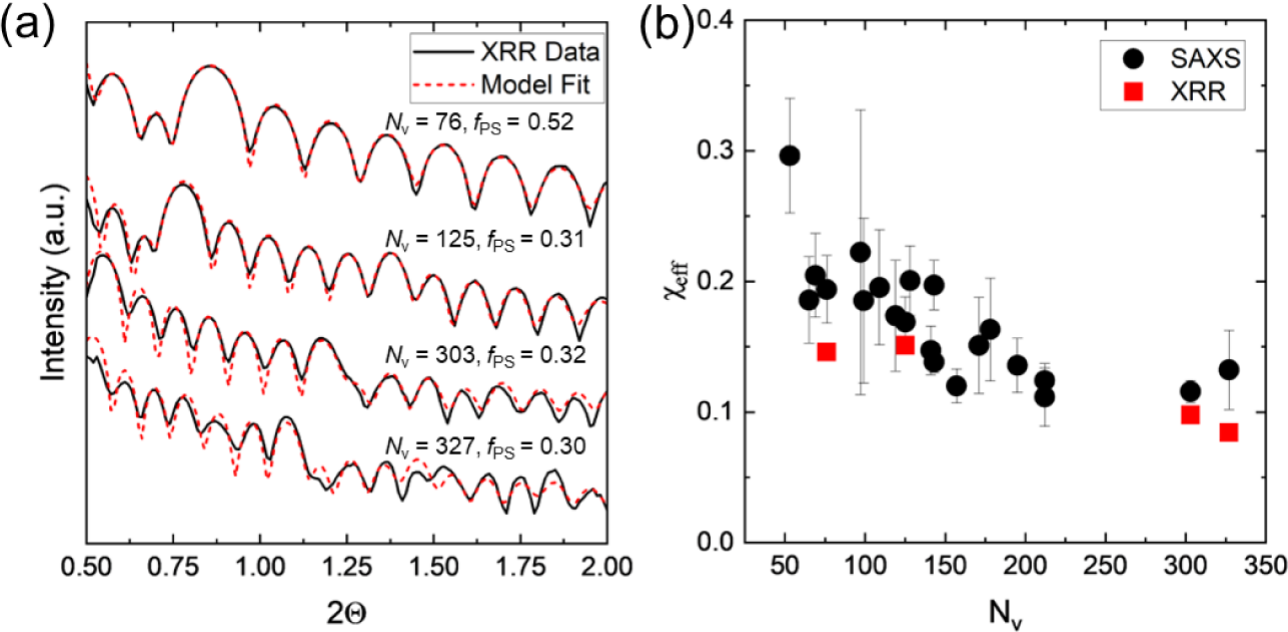
(a) XRR data and model fits and (b) χ_eff_ values, determined using interfacial width and domain spacing values from SAXS and XRR measurements and calculated using SST, as a function of block copolymer repeat unit length, represented by *N*_V_.

### Modeling of Domain Spacings and Estimation of χ Using SST

Although SST only models the interfacial widths well at the higher values of *N*_V_ studied, its predictions for the domain spacing rely only on the less restrictive assumption of large χ_eff_ *N*_V_ values and could therefore extend to lower values of *N*_V_ given the high incompatibility of the blocks in this PS-*b*-PPFS system. It is also possible that the domain spacings at lower *N*_V_ values could be modeled by treating very short blocks as rods rather than coils. In our data, there is a cluster of several points at low *N*_V_ that have short PPFS blocks, and there is also evidence in the literature that fluorination in the side chain can lead to an increase in rigidity.^62^ Therefore, we model these low-*N*_V_ points using a rod–coil approach with the PPFS block as the rod. Next, we use information from the high-*N*_V_ coil–coil and the low-*N*_V_ rod–coil fits to estimate the difference in stiffness of the two blocks.

Figure 4a shows the domain spacing of the lamellae versus *N*_V_, which is measured in units of the segment volume of PS, 1/ρ_0PS_. At higher values of *N*_V_, this log–log plot shows the standard behavior for coil–coil polymers,^6^ with the points lying close to a straight line with a slope of approximately 2/3. However, below *N*_V_ ≈ 120, the slope of the data becomes shallower, in contrast to the standard behavior of coil–coil block copolymers in both experiment^63,64^ and theory,^65,66^ wherein the slope of the curve becomes steeper at low *N*_V_ values.

**Figure 4:**
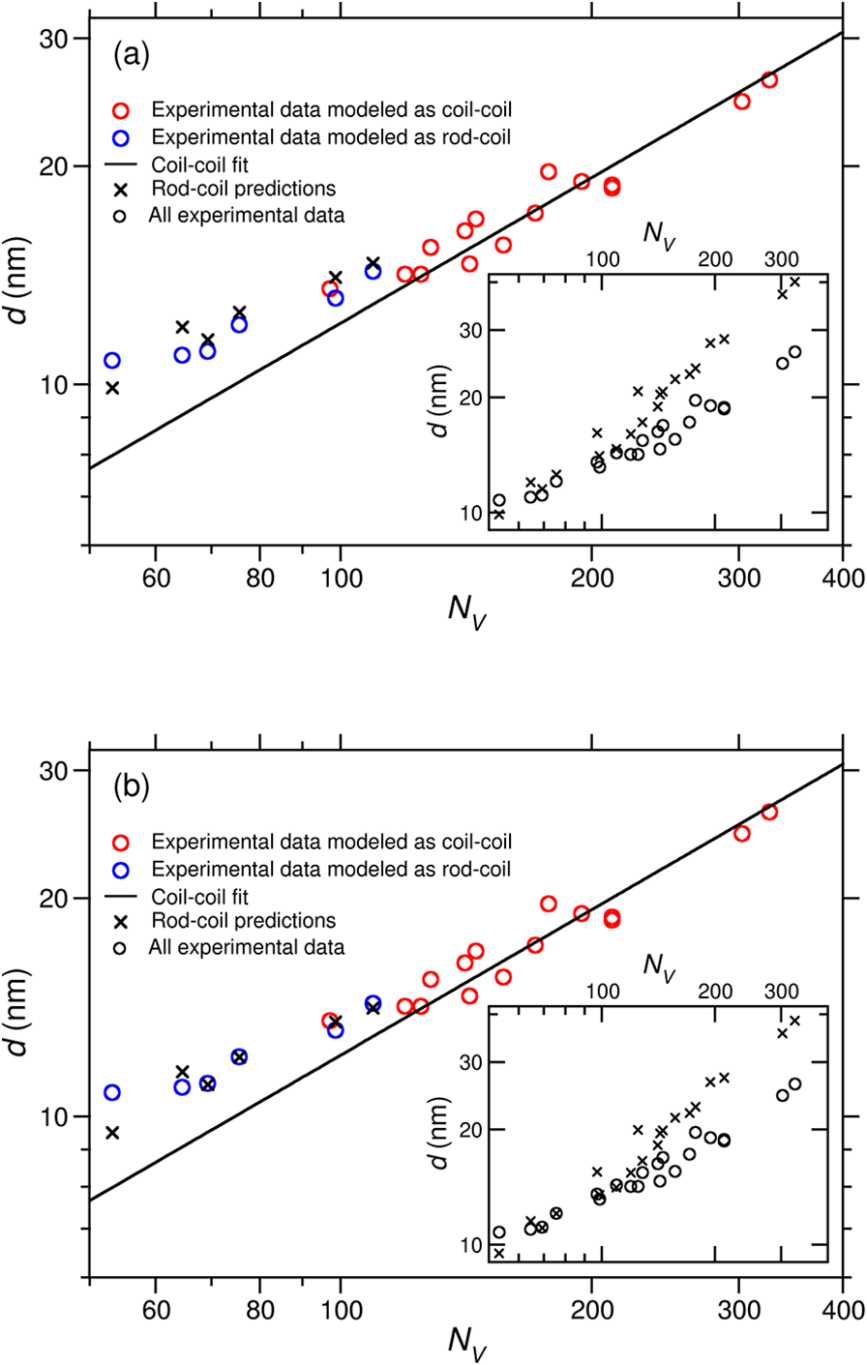
(a) *d* in the lamellar phase vs *N*_V_. The circles show experimental results; those in red have been fitted by a coil–coil SST formula (eq 5), and the spacing of the blue points has been predicted using a rod–coil SST model (eq 4). The solid line shows the results of a coil–coil fit (with a slope of 2/3 on the log–log plot), and the black crosses show the predictions of the rod–coil model. The inset displays the same experimental data and includes the predictions of the rod–coil model at higher *N*_PPFS_ values. (b) As in part (a) with the predictions of the rod–coil model shifted by an alternative calculation of the prefactor.

The domain spacing data were fitted in the region wherein standard coil–coil behavior was observed using SST.^37^ As discussed above, molecules with a shorter PPFS block are modeled as rod–coil.^45^ The close relationship between the two models allows the parameter determined by the SST fit to be used in the rod–coil model to predict the domain spacings for shorter PPFS blocks.

To begin, it was assumed that, provided the coil–coil picture is valid, the greater stiffness of PPFS is partly compensated for by its larger segment volume (ρ_0PS_/ρ_0PPFS_ ≈ 1.26) so that the conformational asymmetry is low, ϵ ≈ 1, and eq 3 reduces to the original SST prediction^37^ of

5 $$d\approx 2{\left(\frac{4\gamma a_{PS}^{2}}{\pi^{2}\rho_{0PS}}\right)}^{1/3}N_{V}^{2/3}$$

Even if ϵ deviates somewhat from 1, the quantity (ϵ*f*_PPFS_ + 1 – *f*_PPFS_)^−1/3^ will remain close to 1 due to a degree of cancellation between the ϵ*f*_PPFS_ and *f*_PPFS_ terms in eq 3 and the low power (−1/3) to which this factor is raised. The data points for which the coil–coil picture is more appropriate were subsequently fitted with eq 5. It is somewhat arbitrary which points should be included in this fit, and, for definiteness, the cluster of six samples with shorter PPFS blocks (*N*_PPFS_) between 20 and 29 was excluded from the coil–coil fit (these points are modeled later with the rod–coil theory), and all other domain spacing data were fitted with the coil–coil formula (eq 5). The agreement between the coil–coil model and the experimental data was good (Figure 4a), and it was demonstrated that using a different selection of points (specifically, excluding the 10 samples with *N*_PPFS_ ≤ 53 from the coil–coil fit) had little effect on the fit (see Figure S8), giving us confidence in our approach.

It was then assumed that the coil–coil model breaks down for shorter PPFS blocks and that a rod–coil theory would be more appropriate. In the sample with the longest PPFS block to be treated as a rod (*N*_PPFS_ = 29), the size of the PPFS domain was measured by XRR to be 5.3 nm. If the Kuhn length of PPFS is significantly greater than that for PS (1.8 nm),^67^ it will be not far from the size of a single PPFS layer for all six samples modeled as rod–coil, making this approach reasonable as a first approximation, especially given that *N*_PS_ > *N*_PPFS_ in all but one case, making it appropriate to treat the PS block as a coil.

The prefactor $2{\left(4\gamma a_{\mathrm{PS}}^{2}/\left(\pi^{2}{\rho_{0}}_{\mathrm{PS}}\right)\right)}^{1/3}$ from this fit is then used in eq 4 to predict the remaining *d* values (Figure 4a). In a clear demonstration of a crossover between coil–coil and rod–coil behavior, the agreement with the experimental results was good, and the shallower slope was reproduced well, although the theoretical predictions were, in general, slightly too high. The reason that the predicted points do not lie on a smooth curve is that *f*_PPFS_ varies from sample to sample. The predictions of the rod–coil model for samples with higher *N*_PPFS_ values are shown in the inset to Figure 4a, and it can be seen, as expected, that the rod–coil picture becomes less valid as the length of the PPFS block increases. In particular, the point at *N*_V_ ≈ 97, excluded from the initial set of “rod–coil” points due to its relatively long PPFS block (*N*_PPFS_ = 46) is predicted to have a spacing that is significantly higher than the experimental value. This overprediction is consistent with the idea that its PPFS block has become too long for a rod conformation to be valid. This argument also holds for the point at *N*_V_ ≈ 125, for which the value predicted by the rod–coil formula again lies far above the experimental value. As in the case of the *N*_V_ ≈ 97 point, this sample has a value for *N*_PPFS_ significantly greater than the points on either side (68 vs. 36 and 43), meaning the PPFS block is sufficiently long to adopt a coil conformation and thus approximation as a rod is less appropriate.

As noted above, the trend is reproduced well by the model, but, in general, the predicted values are too high. A small refinement can be made to the calculation by estimating the numerical value of the factor (ϵ*f*_PPFS_ + 1 – *f*_PPFS_)^−1/3^ and using the result to modify the prefactor calculated from the coil–coil fit. To do this, it was assumed that *a*_PS_/*a*_PPFS_ ≈ 0.8, which is close to the corresponding ratios for polyethylene/polytetrafluoroethylene (wherein the difference is fluorination of the polymer backbone) and poly­(methyl methacrylate)/poly­(1,1-dihydroperfluorooctyl methacrylate) (wherein the difference is additional fluorocarbon functionality in the side chain), estimated from simulations^68^ and experiments, respectively.^62^ As anticipated earlier, the factor does not vary strongly for the values of *f*_PPFS_ in the samples that have been fitted and remains in the interval 1.026 ≲ (ϵ*f*_PPFS_ + 1 – *f*_PPFS_)^−1/3^ ≲ 1.050. The average value for these samples is 1.041, and the quantity $2{\left(4\gamma a_{\mathrm{PS}}^{2}/\left(\pi^{2}{\rho_{0}}_{\mathrm{PS}}\right)\right)}^{1/3}$ was then calculated from the prefactor found by fitting the “coil–coil” domain spacing data with $d\propto N_{V}^{2/3}$ by dividing it by 1.041. The result for $2{\left(4\gamma a_{\mathrm{PS}}^{2}/\left(\pi^{2}{\rho_{0}}_{\mathrm{PS}}\right)\right)}^{1/3}$ was then used in the rod–coil formula (eq 4), and the results of this calculation are shown in Figure 4b. All “rod–coil” points in the main figure apart from the leftmost point (*N*_V_ ≈ 53, where both blocks may have become rod-like) now lie closer to the experimental data, and it can be seen from the inset that good agreement is found between the rod–coil formula and the data until *N*_V_ ≈ 130, apart from the *N*_V_ ≈ 97 and *N*_V_ ≈ 125 points noted earlier.

A rough estimate of χ can be found using the quantity $2{\left(4\gamma a_{\mathrm{PS}}^{2}/\left(\pi^{2}{\rho_{0}}_{\mathrm{PS}}\right)\right)}^{1/3}$ determined via the coil–coil fits. This includes the surface tension (γ), which is related to χ in SST by γ = (χ/6)^1/2^ *a*ρ_0_,^37,69^ wherein *a* is the statistical segment length, ρ_0_ is the segment density, and *γ* is measured in units of *k*_B_ *T*. Taking the value of *a* to be that for PS,^61^ so that *a* = *a*_PS_ ≈ 0.67 nm, the Flory–Huggins parameter can be estimated as χ≈ 0.20 (the segment density ρ_0PS_ cancels during the calculation and its value need not be specified). If the prefactor is adjusted as before by using the factor (ϵ*f*_PPFS_ + 1 – *f*_PPFS_)^−1/3^, the estimate is modified to χ ≈ 0.16. It is important to note that the SST calculation that relates γ to χ works on different assumptions to the main domain spacing calculation, and is more accurate with a relatively low value of χ and a high value of *N*_V_ instead of simply a high value of the product χ*N*_V_.^38,69^ Although these conditions are not strictly met here, both these values for χ are consistent with a simple estimate obtained by taking the shortest polymer as being at the order–disorder transition (wherein χ*N*_V_ ≈ 10.495) and calculating χ as 10.495/*N*_V_ ≈ 0.2. The values are also consistent with that found in closely related SST calculations performed on the scattering data (see earlier), even though the treatment of the interface is simpler in the current calculations. The simpler model is used here because the more detailed model^38^ (including finite-*N*_V_ corrections used to fit the scattering data) does not give an absolute value for the surface tension that can be substituted into the prefactor of the formula for the domain spacing.

We have attempted to take the difference in segment lengths into account in detail in the main coil–coil SST calculation. However, apart from at lower values of *N*_PPFS_, there is no connection between the scatter in the experimental data and the scatter predicted by eq 3 with a longer persistence length for PPFS (so that ϵ < 1), and it is not possible to go beyond the current approach at present.

Taking into account the distinct approaches used to estimate χ using SST outlined in this section, we proposed an estimated χ value of 0.2 to represent the thermodynamic incompatibility of the PS and PPFS blocks in this system. This χ value is high compared to many commonly studied AB diblock copolymers such as polystyrene-*b*-poly­(methyl methacrylate) with a χ value at 25 °C of 0.043.^20^ Furthermore, it is possible that the true value of χ is higher than this estimate. In all the calculations of χ presented here, mean-field assumptions are used, in line with the prevailing literature. In experimental phase diagrams,^14^ the value of χ at the order–disorder transition can be shifted to significantly higher values than the mean-field estimate of 10.495 due to fluctuations,^14,70^ with the result that any estimate of χ based on this value will also be increased. In the current system, where rod–coil behavior is seen at lower *N*_V_, this increase may be partially offset by the relatively small shift of the ODT to lower values of *χN*_V_ predicted by rod–coil models,^46^ but the overall effect is likely to be an increase in the value of χ above the mean-field estimate.

### Experimental Phase Diagram

Using the χ value of 0.2 determined using SST approaches, an experimental phase diagram of χ*N*_V_ *versus f*_PS_ was constructed to map the microphase separation behavior of PS-*b*-PPFS block copolymers synthesized in this study (see Figure 5). This experimental phase diagram aligns with classical predicted phase diagrams for AB diblock copolymers reported widely in the literature,^7^ with large phase space occupied by lamellar morphologies, underneath which exists a narrow phase space for hexagonally packed cylinders, then a broad space occupied by a disordered morphology at low χ*N*_V_ values. While we did not observe spherical or gyroid morphologies in the PS-*b*-PPFS samples prepared in this study, we expect such phases could exist for this system outside the mapped samples. The low degree of polymerization of the polymers in the current study might be expected to lead to strong fluctuation effects, giving nearly vertical boundaries between the phases.^14,70^ From this point of view, the agreement of our results with the classical mean-field phase diagram, and specifically the existence of the region of hexagonally packed cylinders below the lamellar region on the left of the phase diagram, could be seen as surprising. A possible explanation for this is that, when *f*_PS_ and *N*_V_ are both small, which is the case in the HEX region on the left of the phase diagram, the PS block has itself become so short that the polymers behave as rod–coil molecules, with PS now acting as the rod. This could lead to the appearance on the lower left of the phase diagram of the broader HEX region, passing below the lamellar region, predicted by rod–coil models^46^ and seen in the current data. Practically, construction of such a phase diagram enables reproducible targeting of specific bulk PS-*b*-PPFS morphologies that may be useful for a range of applications including nanolithography^17,18^ and membrane technologies^19^ such as water filtration,^71^ drug delivery,^72^ and energy applications.^73^

**Figure 5:**
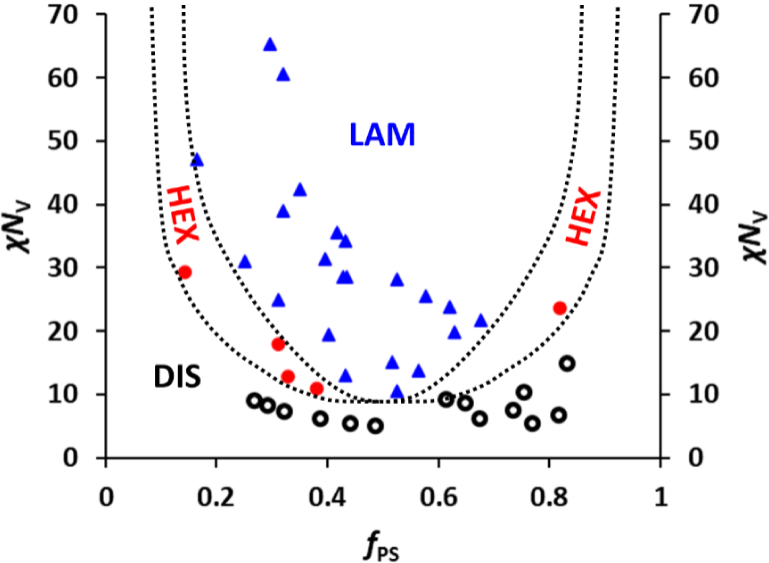
Experimental phase diagram of χ*N*_V_ vs *f*_PS_ constructed for the bulk self-assembly of PS-*b*-PPFS block copolymers. Open black circles represent copolymers with a DIS morphology, filled red circles represent those with a HEX morphology, and filled blue triangles represent those with a LAM morphology. Dotted lines represent proposed phase boundaries. It is important to note that no spherical morphologies were observed between the HEX and DIS regimes for asymmetric block copolymers, but we cannot eliminate their existence.

## Conclusions

A large series of PS-*b*-PPFS block copolymers was synthesized via a two-step RAFT polymerization process in toluene and subsequently purified to yield bulk block copolymers. The resulting block copolymers exhibited *N*_V_ values ranging from 25 to 327, *f*_PS_ values of 0.14 to 0.83 and *Đ* *D* values between 1.07 and 1.32, as determined through NMR spectroscopy and GPC analyses, confirming the generation of a range of well-defined PS-*b*-PPFS samples. The microphase separation of these block copolymers was assessed using SAXS and TEM analyses on thermally annealed samples, which indicated the formation of well-defined morphologies, either lamellae or hexagonally packed cylinders, with multiple peaks observed in the SAXS patterns. The χ value for PS-*b*-PPFS was then estimated to be 0.2 at 22 °C by applying the strong segregation theory to both SAXS and XRR data using calculated *d* and *t*_i_ information, respectively. A crossover from coil–coil to rod–coil behavior driven purely by reducing the length of the fluorinated block and not requiring changes in temperature or chemical composition was demonstrated, and our results are compatible with a moderate difference in rigidity between PS and the stiffer PPFS block. A detailed experimental phase diagram was constructed for bulk PS-*b*-PPFS self-assembly, enabling the reproducible targeting of the desired nanomorphology. Moreover, this formulation enables the generation of high χ-low *N* diblock copolymers despite the relatively similar chemical composition of the constituent blocks, with a clear transition from rod–coil to coil–coil segregation behavior.

## Supplementary Material



## Notes

Sample summary table, polymer characterization including NMR spectroscopy, gel permeation chromatography, thermogravimetric analysis, small-angle X-ray scattering and X-ray reflectivity, and modeling summary discussion (PDF)
