# Supplementary material for: Exploring Microphase Separation in Semi-Fluorinated Diblock Copolymers: A Combined Experimental and Modeling Investigation
Source: ACS Polym Au. 2025 Oct 10;5(6):919–29. doi: 10.1021/acspolymersau.5c00109 (PMC12874174; doi:10.1021/acspolymersau.5c00109)
Supplement: Supplementary file 1 [file lg5c00109_si_001.pdf]

Supporting Information for:

Exploring Microphase Separation in Semi-Fluorinated Diblock Copolymers: A Combined Experimental and Modeling Investigation

Mona Semsarilar,[a,*] Martin J. Greenall,[b,*] Alex H. Balzer,[c,d] Amit Kumar Sarkar,[e] Chaimaa Gomri,[a] Belkacem Tarek Benkhaled,[a] Anke-Lisa Höhme,[f] Martin Held,[f] Volker Abetz,[f] Helena J. Hutchins-Crawford,[e] Georgia L. Maitland,[e,g] Anisha Patel,[e] Thomas H. Epps, III,[c,d,h] Paul D. Topham,[e,g] Matthew J. Derry[e,g,*]

[a] Institut Européen des Membranes (IEM), Univ Montpellier, CNRS, ENSCM, Montpellier, 34090 France.

[b] School of Engineering and Physical Sciences, University of Lincoln, Brayford Pool, Lincoln, LN6 7TS, UK.

[c] Center for Plastics Innovation (CPI), University of Delaware, Newark, Delaware 19716, United States.

[d] Department of Chemical and Biomolecular Engineering, University of Delaware, Newark, Delaware 19716, United States.

[e] Department of Chemical Engineering and Biotechnologies, College of Engineering and Physical Sciences, Aston University, Birmingham, B4 7ET, UK.

[f] Helmholtz-Zentrum Hereon, Institute of Membrane Research, Max-Planck-Straße 1, 21502, Geesthacht, Germany.

[g] Aston Institute for Membrane Excellence, Aston University, Birmingham, B4 7ET, UK.

[h] Center for Research in Soft matter and Polymers (CRiSP), University of Delaware, Newark, Delaware 19716, United States.

Table of Contents

Sample summary 3
Polymer characterization 6
Diblock copolymer phase separation 11
Modeling summary 13
References 13

Sample summary

Table S1. Summary of all polymers synthesized in this study.

Target composition	Monomer conversion (%) [a]	Actual composition [b]	M_n (g mol^{-1}) [c]	M_w (g mol^{-1}) [c]	$Đ$ [c]	N [a]	f_{PS} [d]	N_V [e]	d (nm) [f]	Morphology [f]
PS_{20}	73	PS_{12}	350	400	1.14	12	1.00	12	-	-
PS_{40}	50	PS_{21}	2700	2900	1.07	21	1.00	21	-	-
PS_{60}	50	PS_{28}	1600	1800	1.10	28	1.00	28	-	-
PS_{60}	64	PS_{39}	3600	4100	1.15	39	1.00	39	-	-
PS_{100}	57	PS_{62}	5200	5900	1.12	62	1.00	62	-	-
PS_{140}	50	PS_{74}	6500	7100	1.10	74	1.00	74	-	-
PS_{190}	50	PS_{97}	8700	10000	1.16	97	1.00	97	-	-
PS_{12}-$PPFS_{10}$	100	PS_{12}-$PPFS_{10}$	8600	10100	1.17	22	0.49	25	7.12	DIS
PS_{12}-$PPFS_{12}$	98	PS_{12}-$PPFS_{12}$	8600	10300	1.19	24	0.44	27	6.94	DIS
PS_{12}-$PPFS_{15}$	97	PS_{12}-$PPFS_{15}$	10000	12400	1.24	27	0.39	31	7.33	DIS
PS_{12}-$PPFS_{20}$	97	PS_{12}-$PPFS_{20}$	9800	11800	1.19	32	0.32	37	7.75	DIS
PS_{12}-$PPFS_{25}$	90	PS_{12}-$PPFS_{23}$	10700	12600	1.18	35	0.29	41	8.40	DIS
PS_{12}-$PPFS_{35}$	73	PS_{12}-$PPFS_{26}$	11200	13500	1.20	38	0.27	45	8.99	DIS
PS_{21}-$PPFS_{5}$	99	PS_{21}-$PPFS_{5}$	120	140	1.19	26	0.77	27	5.71	DIS
PS_{21}-$PPFS_{8}$	99	PS_{21}-$PPFS_{8}$	550	650	1.17	29	0.68	31	6.28	DIS

PS_{21}-$PPFS_{27}$	97	PS_{21}-$PPFS_{27}$	800	900	1.13	48	0.38	55	9.67	HEX
PS_{21}-$PPFS_{34}$	97	PS_{21}-$PPFS_{34}$	1500	1700	1.11	55	0.33	64	10.5	HEX
PS_{21}-$PPFS_{160}$	63	PS_{21}-$PPFS_{100}$	15300	16700	1.09	121	0.14	147	13.3	HEX
PS_{28}-$PPFS_{5}$	99	PS_{28}-$PPFS_{5}$	440	510	1.17	33	0.82	34	6.73	DIS
PS_{28}-$PPFS_{8}$	99	PS_{28}-$PPFS_{8}$	410	490	1.18	36	0.74	38	7.10	DIS
PS_{28}-$PPFS_{12}$	99	PS_{28}-$PPFS_{12}$	250	300	1.16	40	0.65	43	7.88	DIS
PS_{28}-$PPFS_{15}$	96	PS_{28}-$PPFS_{14}$	430	510	1.18	42	0.61	46	8.43	DIS
PS_{28}-$PPFS_{20}$	99	PS_{28}-$PPFS_{20}$	1000	1200	1.19	48	0.53	53	10.78	LAM
PS_{28}-$PPFS_{30}$	96	PS_{28}-$PPFS_{29}$	1000	1100	1.14	57	0.43	65	11.0	LAM
PS_{28}-$PPFS_{75}$	65	PS_{28}-$PPFS_{49}$	1200	1500	1.22	77	0.31	90	14.8	HEX
PS_{39}-$PPFS_{10}$	100	PS_{39}-$PPFS_{10}$	1600	2000	1.23	49	0.76	52	7.75	DIS
PS_{39}-$PPFS_{25}$	94	PS_{39}-$PPFS_{24}$	6300	8300	1.32	63	0.56	69	11.1	LAM
PS_{39}-$PPFS_{30}$	95	PS_{39}-$PPFS_{29}$	6300	8000	1.26	68	0.52	76	12.1	LAM
PS_{39}-$PPFS_{50}$	91	PS_{39}-$PPFS_{46}$	7000	9000	1.30	85	0.40	97	13.5	LAM
PS_{39}-$PPFS_{90}$	76	PS_{39}-$PPFS_{68}$	7300	9300	1.27	107	0.31	125	14.2	LAM
PS_{39}-$PPFS_{150}$	61	PS_{39}-$PPFS_{92}$	8400	11000	1.30	131	0.25	155	16.9	LAM
PS_{39}-$PPFS_{400}$	39	PS_{39}-$PPFS_{156}$	9900	13000	1.30	195	0.17	236	16.9	LAM
PS_{62}-$PPFS_{10}$	100	PS_{62}-$PPFS_{10}$	2500	2700	1.26	72	0.83	75	9.52	DIS
PS_{62}-$PPFS_{30}$	97	PS_{62}-$PPFS_{29}$	7500	9700	1.29	91	0.63	99	13.1	LAM

PS_{62}-$PPFS_{75}$	85	PS_{62}-$PPFS_{64}$	7700	9200	1.19	126	0.43	143	14.6	LAM
PS_{62}-$PPFS_{90}$	83	PS_{62}-$PPFS_{75}$	8600	11100	1.28	137	0.40	157	15.6	LAM
PS_{62}-$PPFS_{100}$	66	PS_{62}-$PPFS_{66}$	8400	10800	1.29	128	0.43	143	16.9	LAM
PS_{62}-$PPFS_{200}$	52	PS_{62}-$PPFS_{105}$	9300	12300	1.32	167	0.32	195	19.0	LAM
PS_{74}-$PPFS_{30}$	93	PS_{74}-$PPFS_{28}$	8400	10400	1.24	102	0.68	109	14.3	LAM
PS_{74}-$PPFS_{40}$	91	PS_{74}-$PPFS_{36}$	8500	10200	1.20	110	0.62	119	14.2	LAM
PS_{74}-$PPFS_{68}$	63	PS_{74}-$PPFS_{43}$	7800	9000	1.16	117	0.58	128	15.4	LAM
PS_{74}-$PPFS_{68}$	78	PS_{74}-$PPFS_{53}$	8100	9500	1.16	127	0.53	141	16.3	LAM
PS_{74}-$PPFS_{100}$	77	PS_{74}-$PPFS_{77}$	9000	12000	1.28	151	0.43	171	16.8	LAM
PS_{74}-$PPFS_{140}$	59	PS_{74}-$PPFS_{82}$	12000	14000	1.12	156	0.42	178	19.6	LAM
PS_{74}-$PPFS_{350}$	31	PS_{74}-$PPFS_{109}$	9200	11000	1.21	183	0.35	212	18.6	LAM
PS_{97}-$PPFS_{19}$	90	PS_{97}-$PPFS_{17}$	9800	12300	1.25	114	0.82	118	12.8	HEX
PS_{97}-$PPFS_{170}$	68	PS_{97}-$PPFS_{163}$	13100	16000	1.22	260	0.32	303	24.5	LAM
PS_{97}-$PPFS_{300}$	44	PS_{97}-$PPFS_{182}$	14700	18100	1.23	279	0.30	327	26.3	LAM

[a] Determined using ^{1}H NMR spectroscopy by comparing the signals for unreacted monomer with those from the synthesized polymer.

[b] Determined using ^{1}H NMR spectroscopy by end group analysis (for PS) and using monomer conversion data (for PS-*b*-PPFS).

[c] Determined using THF GPC against PS standards.

[d] Calculated using the degree of polymerization of each block and the solid-state densities of PS and PPFS at 20 °C.

[e] Calculated using $N_v = N_{PS}/f_{PS}$.

[f] Determined using SAXS (DIS = disordered, LAM = lamellar, HEX = hexagonally packed cylinders).

Polymer characterization

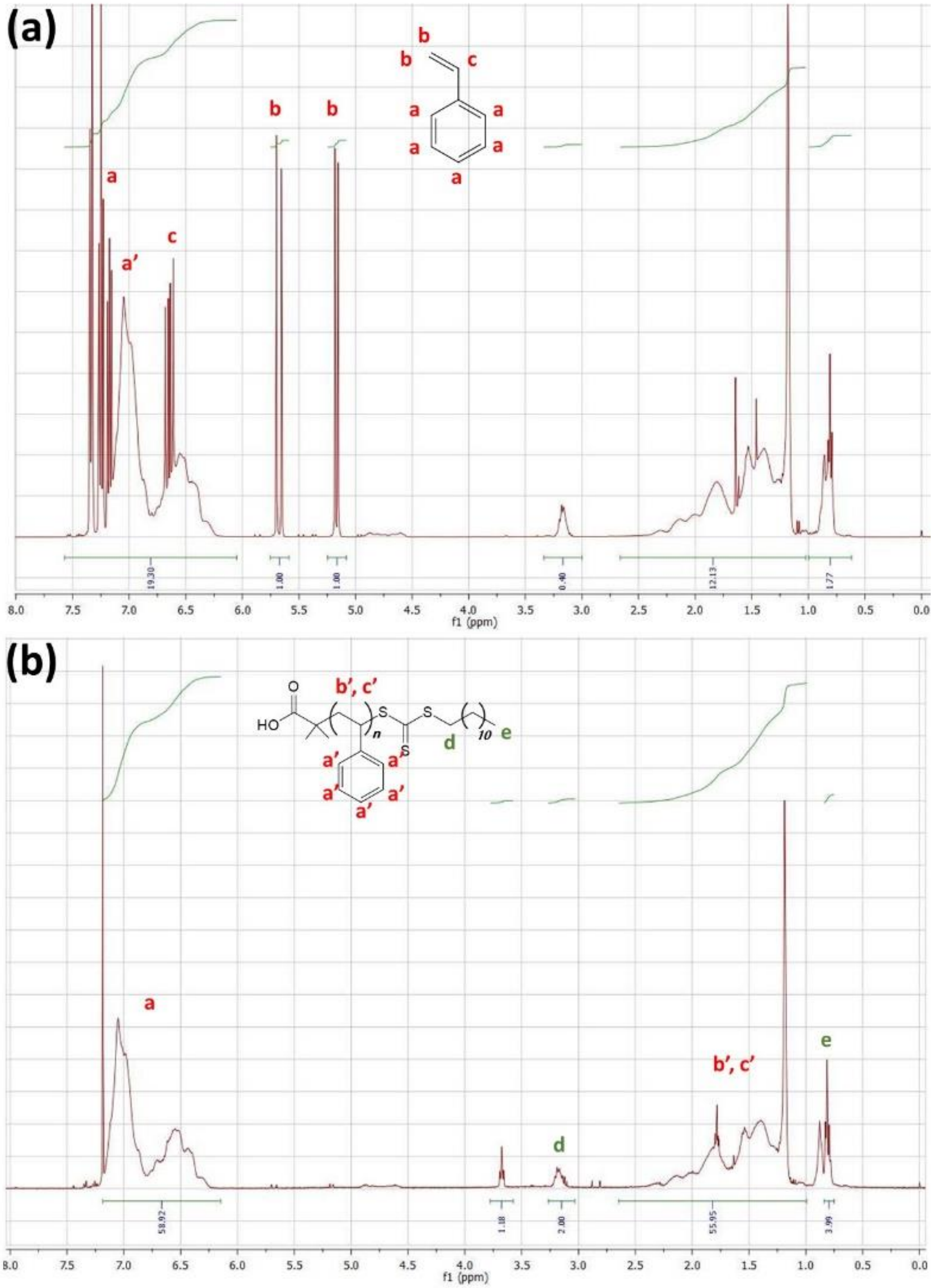

Figure S1. ^{1}H NMR spectrum for (a) crude and (b) purified PS macro-CTA in $CDCl_3$.

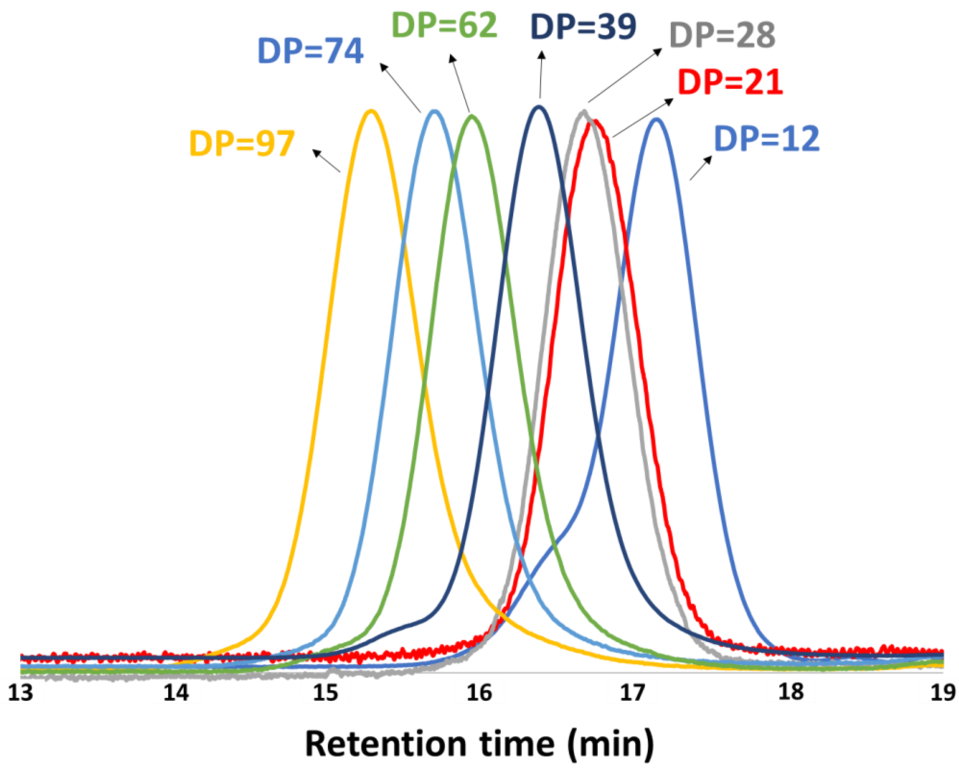

Figure S2. THF GPC data obtained for PS macro-CTAs with varying degrees of polymerization (DPs). Molar mass data obtained against PS standards.

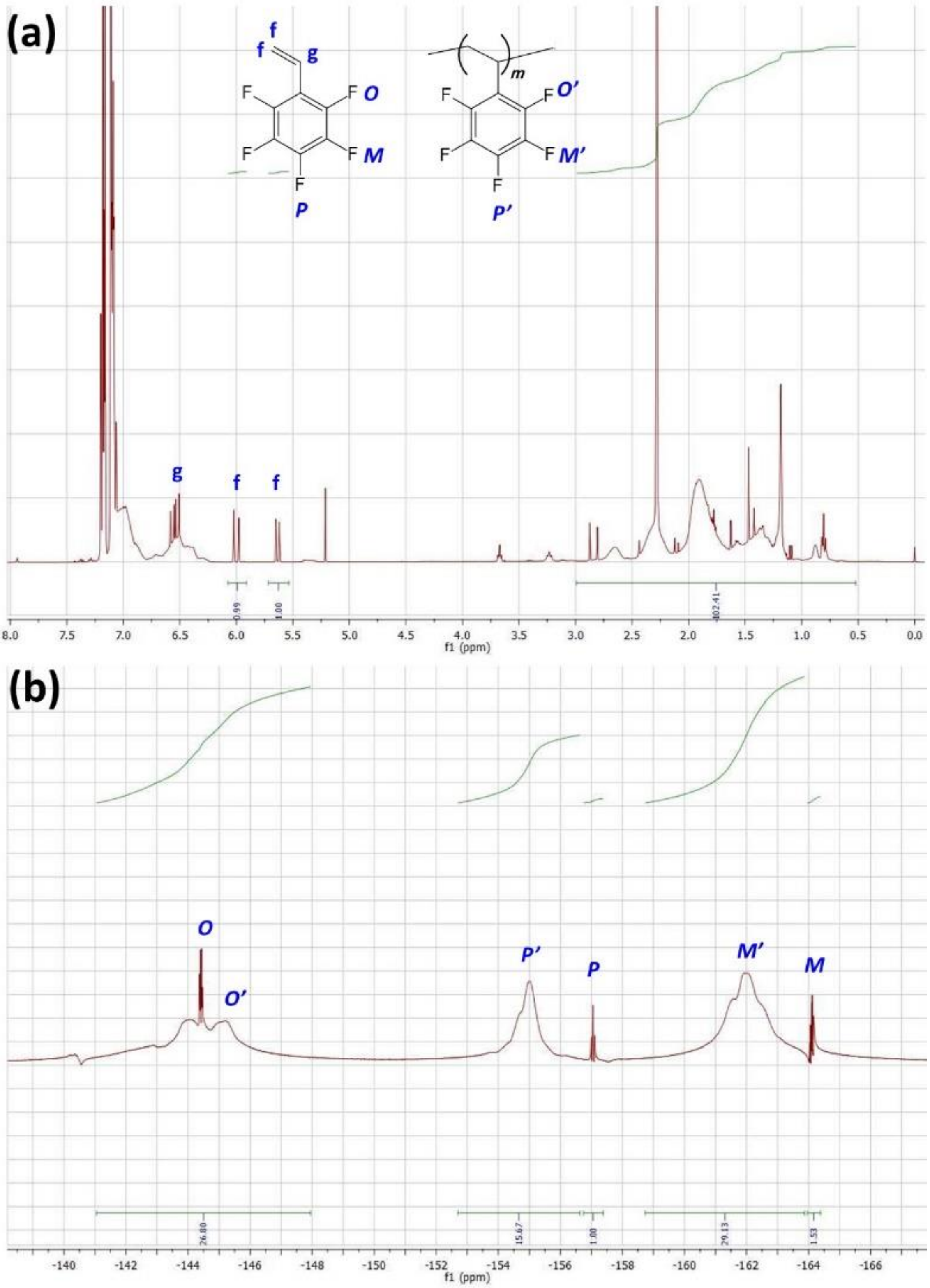

Figure S3. Representative NMR spectra of crude PS-*b*-PPFS in $CDCl_3$: (a) ^1H NMR spectrum and (b) ^{19}F NMR spectrum.

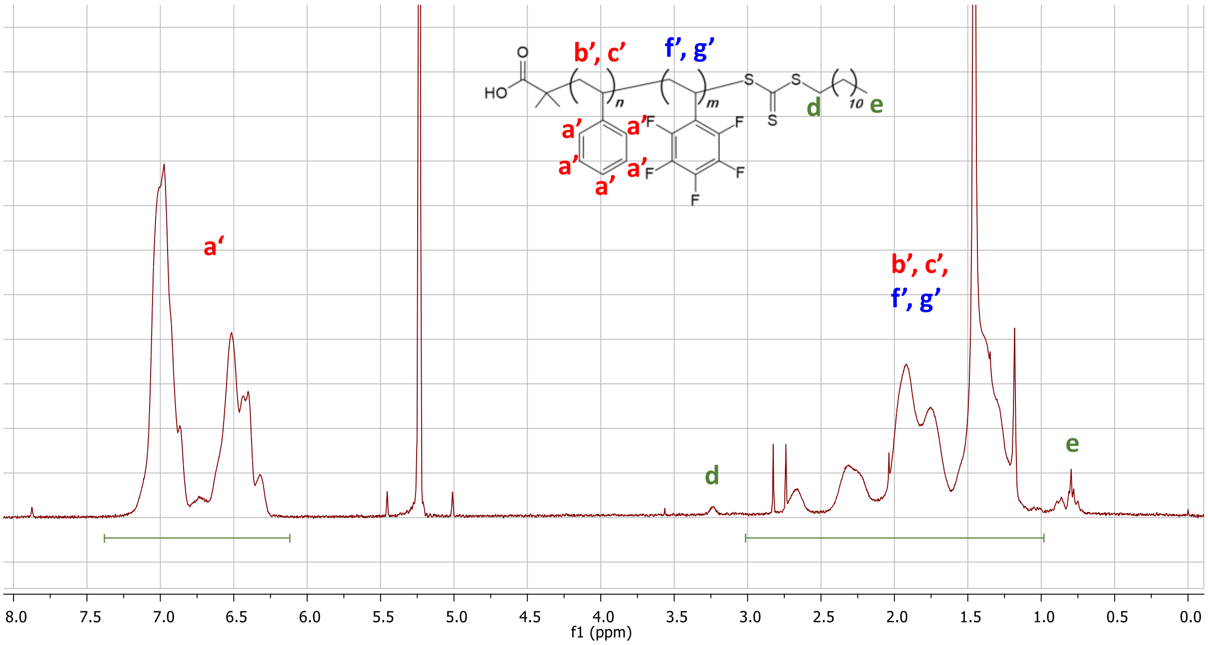

Figure S4. Representative ^{1}H NMR spectrum of purified PS-*b*-PPFS in $CDCl_3$.

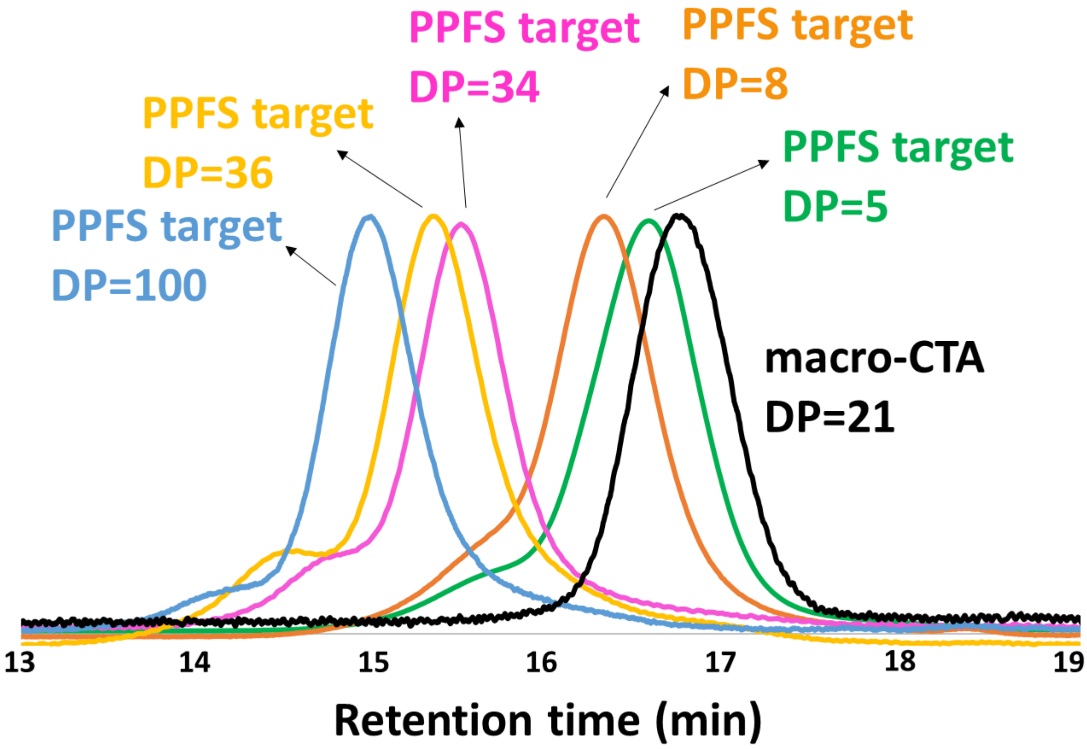

Figure S5. Representative THF GPC data obtained for the series of PS_{21}-*b*-$PPFS_m$ block copolymers, with the DP for the PPFS block noted.

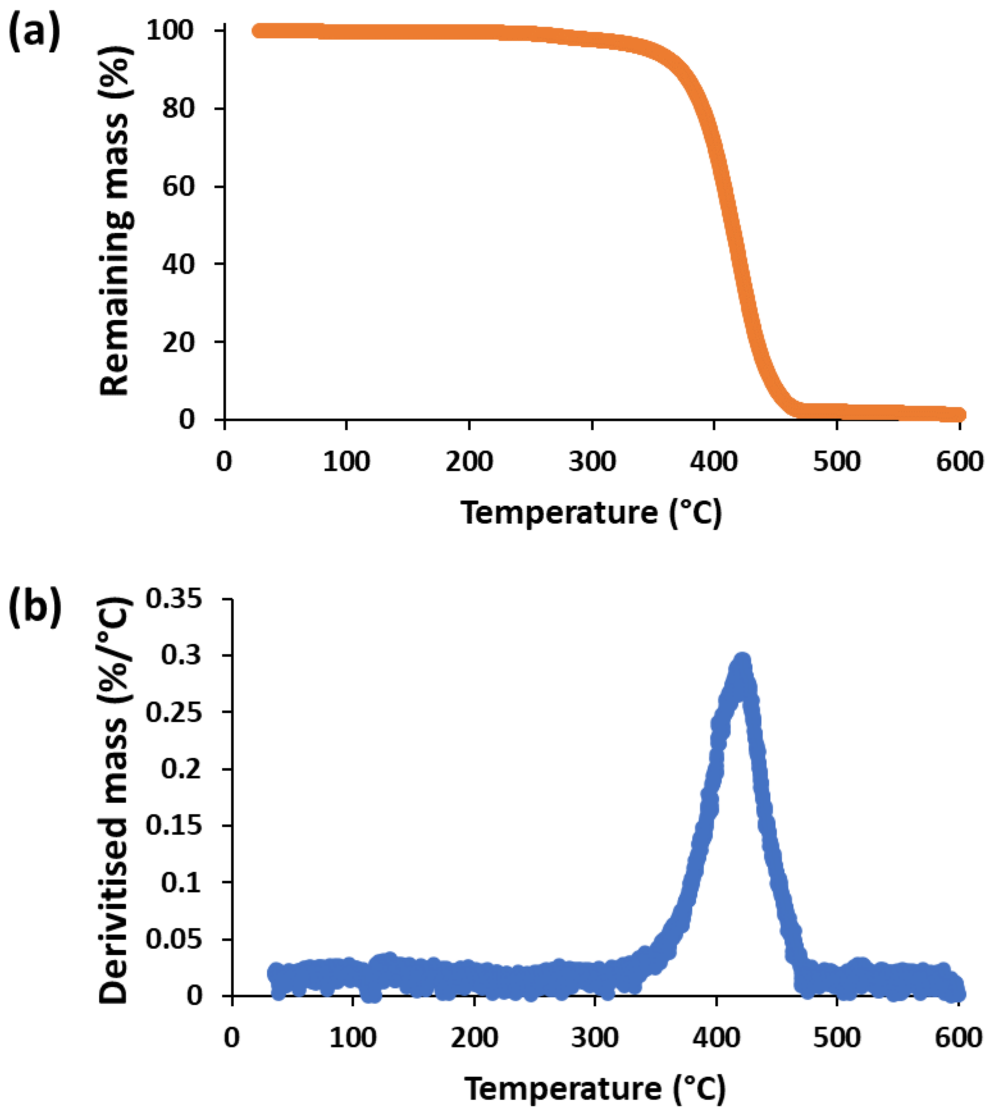

Figure S6. Representative TGA data obtained for PS-*b*-PPFS block copolymers showing temperature profile for (a) remaining mass and (b) the first derivative of the remaining mass.

Diblock copolymer phase separation

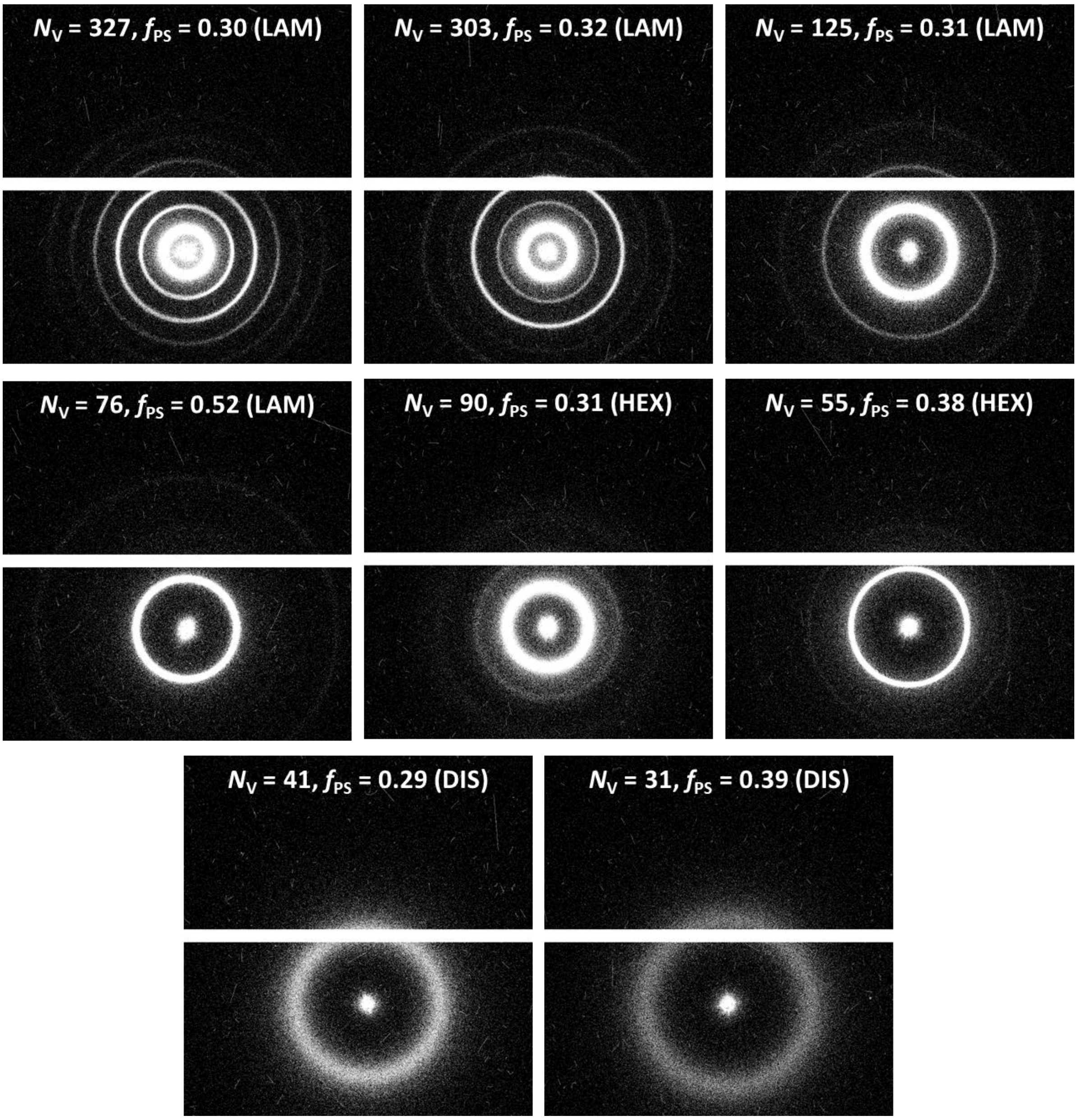

Figure S7. 2D SAXS patterns for selected PS_m-*b*-$PPFS_n$ block copolymers.

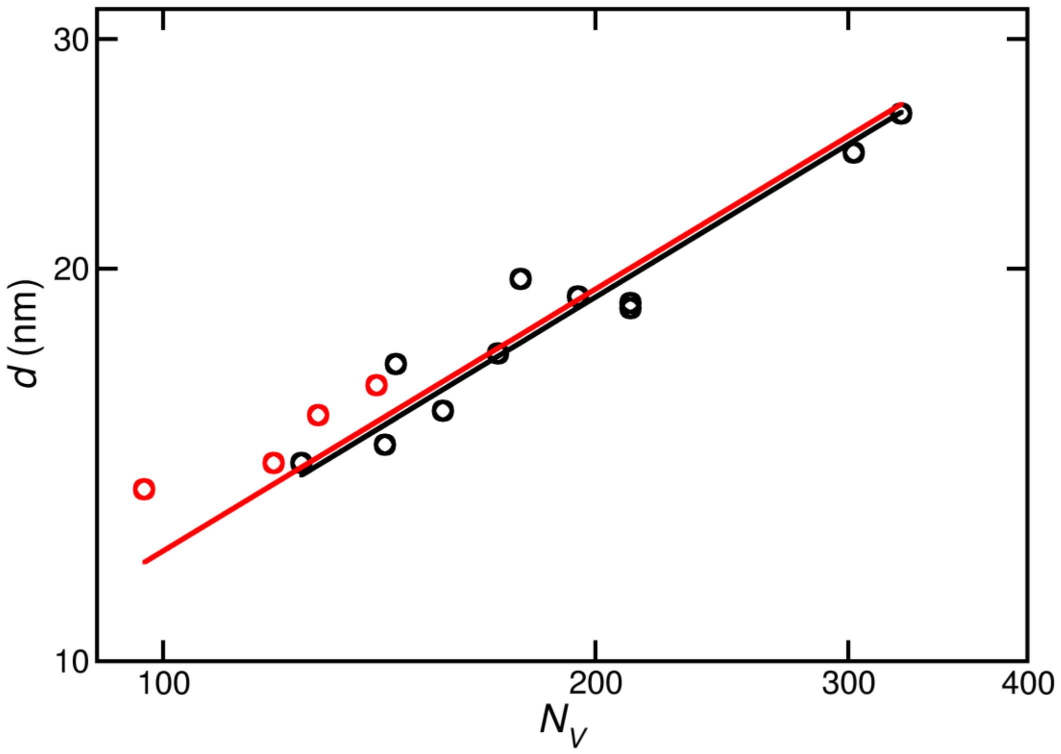

Figure S8. Domain spacing data excluding the sample of six points with N_{PPFS} between 20 and 29 (all open circles) and excluding the sample of ten points with $N_{\mathrm{PPFS}} \leq 53$ (black open circles only). The dashed lines show fits using $d \propto N_V^{2/3}$ to larger (red) and smaller (black) sets of data respectively.

Table S2. Summary of domain spacing (*d*) and interfacial width (t_i) values obtained by SAXS and X-ray reflectometry (XRR).

N_v	f_{PS}	SAXS		XRR	
		***d* (nm)**	**t_i (nm)**	***d* (nm)**	**t_i (nm)**
76	0.52	12.1	1.0	11.6	1.2
125	0.31	14.2	0.9	13.0	0.9
303	0.32	24.5	1.1	22.2	1.1
327	0.30	26.3	1.0	24.9	1.3

Modeling summary

The modeling in the current paper achieves its main goals of estimating χ and demonstrating a crossover between coil-coil and rod-coil behaviour as N_V decreases. However, we note that it would be possible to perform further modeling of the current data by using approaches designed to deal with lower molecular weights. For example, a wormlike chain model[1] could be used, as this is capable of reproducing the curvature of the log-log d vs. N_{V} data noted in the current results at lower N_{V}. Additionally, field-theoretic simulations[2] can provide insights into the effect of fluctuations and finite molecular weights on the value of χ, although they do not, to the best of our knowledge, model rod-coil polymers as yet. Such more detailed modelling might in fact lead to a higher estimate of χ than that made here, as the value of χN_{V} at the order-disorder transition tends to be increased above 10.495 in models that are set up to work with lower values of N_{V}.

References

1. Jiang, Y.; Zhang, X.; Miao, B.; Yan, D.; Chen, J. Z. Y., Microphase Separation of Short Wormlike Diblock Copolymers with a Finite Interaction Range. *Soft Matter* **2016,** *12* (8), 2481-2490.
2. Matsen, M. W.; Willis, J. D.; Beardsley, T. M., Accurate Universal Predictions for Block Copolymer Melts Using Field-Theoretic Simulations. *Macromolecules* **2024,** *57* (9), 4312-4322.
